# Metabolic Control of Epigenetics and Its Role in CD8^+^ T Cell Differentiation and Function

**DOI:** 10.3389/fimmu.2019.02718

**Published:** 2019-11-26

**Authors:** Cansu Yerinde, Britta Siegmund, Rainer Glauben, Carl Weidinger

**Affiliations:** ^1^Division of Gastroenterology, Infectiology and Rheumatology, Medical Department, Charité - Universitätsmedizin Berlin, Berlin, Germany; ^2^Department of Biology, Chemistry and Pharmacy, Freie Universität Berlin, Berlin, Germany; ^3^Clinician Scientist Program, Berlin Institute of Health (BIH), Berlin, Germany

**Keywords:** epigenetics, metabolism, CD8 T cell, exhaustion, anti-tumor immunity, anti-viral immunity

## Abstract

Epigenetic programs that control posttranslational modifications of histone proteins and DNA itself tightly regulate transcriptional networks determining the identity and function of CD8^+^ T cells. Chromatin-modifying enzymes such as histone acetyltransferases and deacetylases, represent key molecular determinants of the epigenetic imprinting of CD8^+^ T cells. The functions of these enzymes highly depend on the availability of key products of cellular metabolism pathways such as acetyl-CoA, NAD (Nicotinamide adenine dinucleotide) and SEM (S-adenosylmethionine), suggesting that there is a close crosstalk between the metabolic and the epigenetic regulation of CD8^+^ T cells. In this review, we will discuss the metabolic regulation of CD8^+^ T cell epigenetics during activation and differentiation. We will furthermore summarize how metabolic signals from the tumor microenvironment (TME) shape the epigenetic landscape of CD8^+^ T cells to better understand the mechanism underlying CD8^+^ T cell exhaustion in anti-tumor and anti-viral immunity, which might help to overcome limitations of current CD8^+^ T cell-based therapies.

## Crosstalk Between Epigenetics and Metabolism

**Figure 1:**
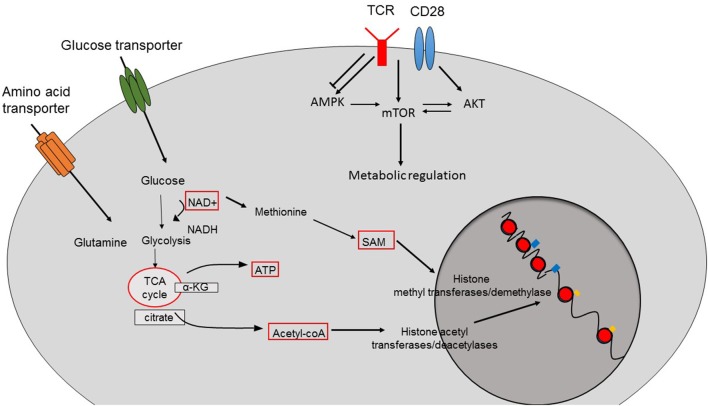
Crosstalk between cellular metabolism and epigenetic changes. The function of epigenetic modifier enzymes depends on the intermediates or the products of cellular metabolism pathways resulting in epigenetic changes and therefore the transcriptional programs of the cells. Acetyl-CoA is the main source for histone acetylation leading to open and permissive chromatin structure. SAM is used by histone methyltransferases and demethylases. The function of sirtuin deacetylases depend on the availability of NAD. TCR-induced activation of pathways such as AMPK, mTOR, and AKT also contributes to the metabolic reprogramming of CD8^+^ T cells.

In order to adapt to shifting environments, CD8^+^ T cells dynamically modulate their transcriptional programs, which not only influence their differentiation but also alter their function and metabolic setup (1). Epigenetic changes are heritable and consist of post-translational modifications of DNA and surrounding histone proteins rather than alterations of primary DNA sequences. In changing external conditions, external stimuli like growth hormones and cytokines activate classical pathways such as mitogen activated protein kinase (MAPK) and nuclear factor of activated T cell (NFAT) signaling resulting in the recruitment, activation or induction of epigenetic modifying enzymes that promote epigenetic alterations in CD8^+^ T cells (2). Similarly, nutrient levels and the metabolic status of CD8^+^ T cells also interfere with the epigenetic programming and subsequently with the function of CD8^+^ T cells (3). Given the fact that epigenetic modifiers harness intermediates or products of key cellular metabolic processes as their cofactors/substrates, regulation of epigenetics by cellular metabolism represents a common biological process (Figure 1) (3), which can disrupt adequate immune responses by CD8^+^ T cells during anti-viral and anti-tumor immune responses (3).

While a wide range of epigenetic mechanisms exists that interfere with the accessibility of the genome by specific transcriptional programs, we will here recapitulate key epigenetic mechanisms and their modifiers as well as their dependency on specific metabolic substrates. Subsequently, we will summarize recent insights into CD8^+^ T cell specific aspects of metabolism-regulated epigenetics in anti-viral and anti-tumor immunity and discuss possible implications of T cell epigenetics for the development of better immunotherapies of cancer.

## Histone Modifications

Eukaryotic DNA is packed in the form of chromatin. Thereby, nucleosomes, the basic unit of the chromatin, consist of 147 bp of DNA, which wraps around the histone octamer composed of two H2A and H2B dimers as well as a tetramer of H3 and H4 proteins. N- and C-terminus of histone tails, which protrude from the nucleosome, represent the main sites for posttranscriptional modifications (PTMs) including acetylation, methylation, phosphorylation and ubiquitination (4). PTMs of histone tails can either directly regulate the chromatin structure, resulting in altered DNA accessibility (5), or can act as platforms for the binding or recruitment of non-histone proteins, known as writers (histone-modifying), readers (histone-modification-recognizing) or erasers (histone modification-erasing) (6). This combination of PTMs on histone tails constitutes the “histone code” that regulates the eukaryotic transcription (5). Histone chaperons are also critical regulators of DNA accessibility since the association of histones with specific chaperons regulates their folding, oligomerization, PTMs or stabilities (5). Therefore, different histone variants contribute to the regulation of DNA accessibility and epigenetic memory (7).

### Histone Acetylation and Deacetylation

Acetylation and deacetylation of histones are among the best-studied epigenetic modifications. Acetylation of lysine residues is catalyzed by histone acetyltransferases (HATs) and reduces their positive charge, therefore the strength of electrostatic interaction between negatively charged DNA, resulting in relaxation of histone-DNA interactions, which leads to an increased accessibility of the DNA for transcription or transcription factors (TFs), respectively (8). Deacetylation reverses this permissive state via condensation of the chromatin structure (9). HATs are classified according to their localization. Cytoplasmic B-type HATs for example participate in the transport of newly synthesized histones from the cytoplasm to the nucleus, while nuclear A-type HATs take control of acetylation events related to the transcription (10). HATs can be further grouped according to their functional motifs consisting of Gcn5-related N-acetyltransferase (GNAT), Moz, Ybf2/Sas3, Sas2, Tip60 (MYST), Creb-binding protein/P300 (CBP/P300) and Rtt109 HAT families (10, 11).

HATs use acetyl-CoA as their primary source for histone acetylation. Acetyl-CoA is a central metabolite and the only source of acetyl groups in the cell (12). Most commonly, acetyl-CoA is produced in the mitochondrial matrix through glycolysis, β-oxidation or the catabolism of branched amino acids (12). As a central metabolite and important signal transducer, acetyl-CoA regulates several cellular processes by controlling the balance between anabolic and catabolic reactions. Therefore, fluctuations in cellular acetyl-CoA levels can also affect the acetylation patterns of histones resulting in varying gene expression and function as well as distinct differentiation programs of cells (12).

Histone deacetylases (HDACs) are responsible for removing acetyl groups from acetylated histones resulting in chromatin condensation and a repressive chromatin structure. Depending on their homology and functions, HDACs are grouped into four different classes: class-I (HDAC1, HDAC2, HDAC3, HDAC8), class-IIa (HDAC4, HDAC5, HDAC7, HDAC9), class-IIb (HDAC6, HDAC10), class-III (Sirt1-Sirt7) as well as class-IV (HDAC11) (13). Although the diversity of the HDAC classes challenge the design of HDAC-inhibitors, several HDAC inhibitors are in clinical use or are under clinical investigation (14). While Vorinostat (SAHA) and Romidepsin (FK288) have been approved for the treatment of cutaneous T-cell lymphoma, Panobinostat (LBH589) and Belinostat (PXD101) are currently used for treating peripheral T-cell lymphoma and multiple myeloma, respectively (14). However, these inhibitors are pan-HDAC inhibitors, therefore studies attempting to design specific HDAC-inhibitors are active areas of research. According to clinical and experimental studies, inhibition of HDACs results in anti-neoplastic effects mostly via cytotoxic and pro-apoptotic mechanisms (15) [e.g., via stabilization of acetylated p53 (16)]. There are also accumulating data proving that inhibition of HDACs in non-oncological settings has important anti-inflammatory effects depending on the cell, tissue and context (15, 17, 18). For example, the gut microbiota-derived short-chain fatty acid butyrate modulates the transcriptional program of CTLs resulting in increased expression of IFNγ and granzyme B (19). However, the effects of butyrate are not mediated by the interaction with its receptors GPR41 and GPR43, but rather through HDAC inhibition resulting in a differential gene expression of CTL effector molecules, which was further validated by pan-HDAC inhibitor treatments (19). HDAC7, which is a Class-IIa HDAC, plays a pivotal role in the regulation of positive and negative selection of thymocytes and immune tolerance as well as their survival (20–22). Serine-threonine phosphoproteome analysis of CTLs by high resolution mass spectrometry revealed that HDAC7 is phosphorylated independently of T-cell receptor (TCR) activation and signaling, leading to its constitutive cytosolic localization (23). The exclusion of HDAC7 from the nucleus is critical for maintaining normal CTL function since the ectopic expression of the nuclear-trapped mutant phosphorylation-defective HDAC7 resulted in lower CD25 expression and subsequently reduced proliferation of CTLs in response to IL-2 (23). However, the role of HDAC7 in adult CD4^+^ and CD8^+^ T cells is still poorly understood. HDAC5, another class-IIa HDAC, has been described as a modulator of the inhibitory functions of Foxp3^+^ regulatory CD4^+^ T cells (Treg) (24), but inoculation of *Hdac5* knockout mice with congenic TC61 lung adeno-carcinoma cells did not result in decreased tumor growth compared to wild type littermates despite a defective immune suppressive capacity of *Hdac5*-deficient Treg, which can be explained by a simultaneous impairment of IFNγ production in *Hdac5*-deficient CD8^+^ T cells (24). The inhibition of HDAC6 (Class-IIb) with its specific inhibitor (ACY-1215) results in impaired proliferation and activation as well as impaired pro-inflammatory cytokine production of CD8^+^ T cells during mouse models of skin inflammation, suggesting that HDAC6 represents a key regulator of TCR-signaling and function, therefore might serve as a new drug target for the treatment of CD8^+^ T cell-related skin disorders (25). On the other hand, the inhibition of HDAC6 in T cells of melanoma patients results in improved anti-tumor capacities of T cells (26). HDAC6 also takes role in the dynamics, transport and secretion of lytic granules to the immune synapse in CD8^+^ T cells, further proving its significance for CD8^+^ T cell function (27). In addition, HDAC3 (Class-I) is required for the proper T cell development in the thymus since its lymphocyte specific deletion resulted in reduced immature CD8 single-positive as well as CD4/CD8 double positive populations (28). Similarly, Class-I HDACs, HDAC1, and HDAC2, also participate in the proper thymic development of T cells (29, 30). Tschismarov et al. further confirm the critical role of HDAC1 during the development of T cells in the thymus. Additionally, they prove that T-cell specific deletion of HDAC1 results in impaired anti-viral responses upon LCMV infection and impaired expansion of LCMV-specific CD8^+^ T cells (31).

Among other HDAC isoforms, Sirtuins (HDAC class-III) were intensively studied in terms of their metabolic functions. They participate in different cellular processes including the regulation of metabolism, DNA repair and mitochondrial function (32). For their deacetylation functions, sirtuins require NAD^+^, which is an essential coenzyme and participating in many redox reactions as in glycolysis, TCA cycle and fatty acid oxidation. Thereby, the provision of NAD^+^ depends on its intracellular compartmentalization, synthesis as well as on metabolic and other pathways that use NAD^+^. For instance, SIRTs were found to be an indirect target of the compound resveratrol leading to histone deacetylation due to increased NAD^+^ availability suggesting that the level of NAD^+^ is critical for the regulation of the epigenome of CD4^+^ T cells through sirtuins (33, 34). Interestingly, human CD8^+^CD28^−^ T cells, which represent a highly cytotoxic population of terminally differentiated memory T cells (Tmem), display an increased glycolytic capacity, which could be linked to a decreased expression of SIRT1 through a forkhead box protein (FOXO1)-dependent manner suggesting that the evolutionary conserved FOXO1-SIRT1 axis is critical for the metabolic reprogramming of human CD8^+^ Tmem (35).

### Histone Methylation

Unlike histone acetylation and deacetylation, which mark the chromatin for either transcriptional activation or repression, the effects of histone methylation on transcription are context dependent. For instance, while tri-methylation of lysine 4 on histone 3 (H3K4me3) triggers transcription, tri-methylation of histone 3 on lysine 27 (H3K27me3) is a sign for condensed chromatin and repressed gene transcription. Depending on the degree of methylation, different groups of histone methyltransferases catalyze the methylation of lysine residues. For example, H3K4 methylation is catalyzed by Set1 methyltransferases, whereas H3K9 methylation is catalyzed by KMT1 methyltransferases as well as H3K27 methylation by enhancer-of-zest homolog-2 (EZH2) (6).

Histone methyltransferases use S-adenosyl-methionine (SAM) as their source for methyl groups (36). Thereby, SAM is produced from methionine via one-carbon metabolism. In immune cells, one-carbon metabolism plays important roles especially in the regulation of proliferation. For instance, Ma et al. showed that once CD4^+^ or CD8^+^ T cells are activated, the expression of genes coding for one-carbon metabolism-related enzymes such as *Shtm1* and *Shmt2*, that are essential regulators of the entry of serine-dependent carbon into the cytosolic and mitochondrial tetrahydrofolate cycle, are up-regulated. Serine that is metabolized through this pathway is required for proper T cell proliferation both *in vitro* and *in vivo* (37).

### ATP-Dependent Chromatin Remodeling Complexes

The formation of higher order chromatin structures is pivotal for the transcriptional programming by regulating or limiting the access of TFs to their binding sites. This structure can be modulated by either PTMs of histone tails or via nucleosome- and chromatin-remodeling complexes. These complexes are capable of removing histones, changing the path of DNA around the nucleosome and hence altering their position. Nucleosome remodeling complexes use the energy generated from ATP hydrolysis (38). Since the activity of these complexes is ATP-dependent, it is expected that fluctuations in cellular ATP levels affect their function, therefore the remodeling of nucleosomes and chromatin structure. However, cellular ATP levels are saturating for their catalytic sites and the activities of chromatin remodeling complexes are not influenced by changes in ATP in the cell. Nevertheless, gene expression states can still be regulated by AMPK signaling which can sense ADP/ATP ratios and induce transcriptional regulation (39). Previously, Blagih et al. showed that both CD4^+^ and CD8^+^ T cells are metabolically adapting in response to limited nutrient levels mediated by AMPK regulated mRNA translation as well as glutamine dependent mitochondrial metabolism. This is a key mechanism for the maintenance of T cell bioenergetics and survival. Their data equally indicated that AMPK signaling is mandatory for primary T cell responses to both, bacterial and viral infections, thus driving adaptive immunity (40). Interestingly, T cell specific deletion of AMPK in mice resulted in increased tumor growth, caused by an impaired tumor killing of CD8^+^ T cells. Deletion of AMPK in T cells resulted in a decreased production of IFNγ and granzyme B as well as an elevated serine/protein phosphatase activity upon activation, resulting in decreased survival rates and anti-tumor functions of CD8^+^ T cells, which could be reversed by inhibition of phosphatase activity (41).

## Metabolic Reprogramming of CD8^+^ T Cell Differentiation and Function

**Figure 2:**
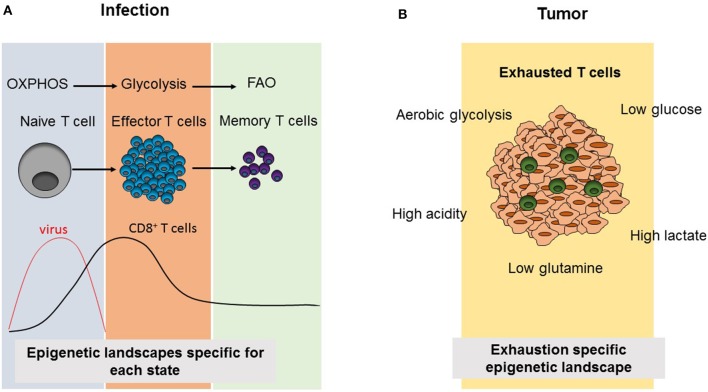
Comparison of CD8^+^ T cell differentiation and metabolism as well as epigenetic landscapes during infection and tumorigenesis. **(A)** Virus infection results in the activation of naïve CD8^+^ T cells triggering the differentiation into effector cells, which induce viral clearance. Subsequently, effector T cells contract and leave behind a small population of memory CD8^+^ T cells. During this differentiation process, CD8^+^ T cell subsets use the indicated cellular metabolism pathways and acquire different epigenetic landscapes specific to each phase. **(B)** In tumors, the presence of immunosupressive environments due to metabolic alterations in tumor cells results in an exhausted phenotype, in which tumor infiltrating T cells are not able to compete with tumor cells for metabolic products and they become non-functional resulting in increasing tumor growth. Exhausted T cells also acquire an exhaustion-specific epigenetic landscape.

In order to adapt to dynamic environments and to meet the demands of cells for their different functions, cellular metabolism is tightly controlled. Cells are capable of performing catabolic and anabolic processes to break down or synthesize macromolecules, which supply either energy in the form of ATP to meet their energy demands, or metabolic intermediate products that are essential for cellular growth (Figure 2A). Via the glycolysis pathway, two molecules of ATP per glucose molecule and pyruvate are produced. In oxygen-rich conditions, pyruvate can enter into tricarboxylic acid (TCA) cycle where it is further processed to generate 38 ATP (maximal number) molecules via oxidative phosphorylation (OXPHOS) (42). Catabolism of pyruvate is not the only mechanism providing substrates for TCA. While fatty acids are converted into acetyl-CoA through fatty acid oxidation (FAO), amino acids are catabolized into 3-, 4-, and 5- carbon substrates that are fed into the TCA cycle (42).

Different metabolic requirements for different cell states are also valid for CD8^+^ T cells. CD8^+^ T cells mainly have three phases as naïve, effector (Teff) and memory T cells. When naïve T cells encounter their antigens, this results in their activation leading to rapid proliferation, growth and differentiation (43). CD8^+^ T cells mostly differentiate into CTLs, producing cytotoxic molecules such as granzyme B, perforin, and pro-inflammatory cytokines including IFNγ and TNFα. Following this effector phase, the effector cell population contracts and a small population of memory T cells (Tmem) persists, which will turn again into CTLs in case of antigen re-challenge and which can persist in the body for years (43). While naïve T cells are metabolically quiescent and depend on OXPHOS, their activation results in a switch into glycolysis pathway to meet the demand for anabolic intermediates necessary for their rapid growth, proliferation and effector functions (Figure 2A). Recently, Store-Operated Calcium Entry (SOCE) signaling, which is the main calcium influx pathway in T cells in response to TCR activation and is mediated by stromal interaction molecule (STIM) 1 and STIM2 as well as ORAI proteins, was shown to control clonal expansion of both, CD4^+^ and CD8^+^ T cells, via controlling glycolysis and OXPHOS through the transcriptional regulation of glycolysis related gene expression (44). It was also shown that these effects are mediated by calcineurin and NFAT, which are the downstream regulators of SOCE (44). Metabolically, Tmem depend mostly on FAO and have a higher spare respiratory capacity (SRC) which supplies the high energy levels needed for their rapid function in case of antigen re-encounter (45–48). However, during most of these studies CPT1α, the rate-limiting enzyme of long-chain FAO was targeted by the drug etomoxir (49, 50). Interestingly, T cell specific deletion of *Cpt1*α *in vivo* proved that CPT1α is dispensable for Teff or Tmem responses as well as CD4^+^ Treg suppressive function, differentiation and hemostasis (50). As the use of the CPT1α inhibitor etomoxir at a concentration higher than 3 μM causes off-target effects, it appears that other metabolic pathways than long-chain FAO are involved in Teff and Tmem differentiation (49, 50).

In conditions of continuous antigen exposure like chronic infections and tumors, T cells fail to differentiate into functional memory cells, but enter a state in which they are hypo-responsive (51). This so-called exhausted cells are incapable of cytokine secretion, proliferation or lysing target cells, paralleled by a sustained expression of co-inhibitory molecules such as PD-1, LAG3 and TIM3 (51). There are several studies, which link the expression of co-inhibitory molecules in T cells with disturbances of metabolic pathways including the PI3K/Akt/mTOR pathway. Thus, Staron et al. recently demonstrated in a mouse model of chronic lymphocytic choriomeningitis mammarenavirus (LCMV) infection, that AKT and mTOR activation are impaired in virus antigen-specific CTLs resulting in a defective anabolic metabolism and enhanced activity of the TF FOXO1 due to its defective phosphorylation and subsequent nuclear trapping (52). Additionally, FOXO1 acts as a direct transcriptional activator of PD-1 as the nuclear localization of FOXO1 promotes the differentiation of terminally exhausted PD-1^hi^Eomes^hi^ CTLs. In contrast, during chronic LCMV infection in mice blockage of PD-1 improves mTOR activity in antigen-specific CTLs while anti-PD-1 treatment was ineffective if mTOR was inhibited by rapamycin (52). Remarkably, the glycolytic metabolism of CD8^+^ T cells is already affected during the acute phase of viral infection in LCMV-infected mice and precedes further dysfunction of antigen-specific T cells suggesting that antigen-specific CD8^+^ T cells are unable to meet the metabolic demands needed for proper cytotoxic function (53). PD-1 is also an early regulator of genes related to glycolysis and mitochondrial function and represses peroxisome proliferator-activated receptor gamma coactivator 1-alpha (PGC1α), whose overexpression is able to improve the metabolism of exhausted T cells and hence, partially restoring their functions (53). These studies suggest that metabolic impairments, which are regulated by PD-1, are early drivers of CD8^+^ T cell exhaustion (53).

The expression of the pro-inflammatory cytokine IFNγ by activated T cells is regulated through 3′ -untranslated region (UTR)-dependent mechanisms (54). Peng et al. showed that in activated T cells the expression of lactate dehydrogenase A (LDHA) is induced in order to support high levels of aerobic glycolysis, but also regulates the expression of IFNγ through 3′-UTR-independent mechanisms. Interestingly, high LDHA levels in activated T cells result in the maintenance of increased acetyl-CoA concentrations leading to increased histone acetylation and facilitating H3K9Ac accumulation on the IFNγ locus, therefore resulting in its increased transcription (55).

Upon TCR-activation, S-2-hydroxyglutarate (S-2-HG) accumulates in murine CD8^+^ T cells up to millimolar concentrations under physiological oxygen conditions through hypoxia inducible factor 1 alpha (HIF-1α) predominating over R-2-hydrooxyglutarate, which is an oncometabolite, produced via mutant isocitrate hydrogenase (IDH) (56). The accumulation of this metabolite results in changes in T cell differentiation, especially resulting in a central memory (Tcm) like phenotype that is stable after transfer into wild-type host mice. The accumulation of S-2-HG also resulted in higher proliferation, maintenance and anti-tumor functions of CD8^+^ T cells *in vivo* following their adoptive transfer into mice (56). Interestingly, these effects were mediated by the modulation of histone and DNA methylation. S-2-HG is an immunometabolite, thus further supporting a metabolism-dependent regulation of T cell epigenetics and functions (56).

## Epigenetic Landscapes During CD8^+^ T Cell Differentiation

Enabled by Assay for Transposase-Accessible Chromatin with high-throughput sequencing (ATAC-seq), which allows the identification of accessible regions of chromatin, the dynamic changes of chromatin as well as accessible TF binding sites were identified in response to acute and chronic LCMV infection during naïve, activated, effector, and memory states of polyclonal or antigen-specific CD8^+^ T cells (57, 58). In these studies, more than 70,000 regions were identified as open (accessible) at least in one of the differentiation state and half of these regions were shared among all states suggesting a CD8^+^ T cell-specific chromatin state. Thus, it was shown that in Tmem cells many regulatory sites are in the open configuration, demonstrating that these cells keep a memory-primed gene expression program that can readily and rapidly be activated in case of a secondary infection (57, 58).

Several TFs were identified to control the fate of CD8^+^ T cells during the differentiation of these cells. For instance, T-box expressed in T cells (Tbet), inhibitor of DNA-bing 2 (ID2), interferon regulatory factor 4 (IRF4), B lypmphocyte induced maturation protein 2 (BLIMP-2), and zinc finger E-box-binding homeobox 2 (ZEB2) are required for the differentiation of Teff cells, whereas T-cell factor 1 (TCF1), eomesodermin (EOMES), inhibitor of DNA-binding protein 3 (ID3), B- cell lymphoma protein 6 (BCL6), and FOXO1 control for memory formation in CD8^+^ T cells (59). However, some of these TFs are not differentially expressed in Teff and Tmem subsets, suggesting that additional mechanisms are involved in controlling the fate decision of T cells. By using a model of bacterial infection in mice, Yu et al. have recently characterized the epigenetic landscapes of naïve, effector, memory precursor and memory CD8^+^ T cells, followed by the prediction of putative TF-binding to accessible chromatin regions in each cell subset (59). In addition, the importance of TFs was ranked in each cell subset via bioinformatic analysis. With this approach, the authors identified and experimentally validated two TFs, Yin Yang 1 (YY1) as well as nuclear receptor subfamily3 group C member 1 (NR3C1) as promoters of effector and memory precursor phenotypes (59).

Other studies applying the ATAC-seq technology demonstrated that also exhausted T cells possess a unique chromatin state (60). During acute models of viral infection in mice, exhausted T cells and effector cells share common accessible chromatin sites. However, additional sites on chromatin open or close during the exhaustion of T cells leading to the expression of PD-1. The treatment of exhausted T cells with anti-PD-1 antibodies resulted in the rescue of gene expression associated with effector functions. However, this treatment failed to fully rescue or reverse the exhaustion-specific chromatin signature as well as the exhaustion specific transcriptional program of exhausted T cells (60).

Although it is now known that exhausted CD8^+^ T cells have a unique chromatin state compared to effector or memory subsets, the mechanisms driving their transcriptional and epigenetic development are poorly understood. Recently, three studies identified the transcription factor thymocyte selection associated high mobility box (TOX) as the main factor promoting CD8^+^ T cell exhaustion by regulating early epigenetic events (61–63). These include decreased accessibility of genes associated with effector T cell differentiation as well as increased accessibility of memory and exhausted phenotype associated genes (62). The expression of TOX is a robust hallmark of exhausted T cells. However, it is transiently expressed at low levels during acute viral infections. In addition, the expression of TOX is essential and sufficient to induce the exhausted T cell phenotype as defined by the marker proteins PD-1, TIM3, LAG3, TIGIT as well as EOMES. Interestingly, initial TOX expression requires NFATc2 and calcineurin signaling, however, the sustained expression of TOX in exhausted CD8^+^ T cells is calcineurin-independent (62). This study suggests that among others, TOX expression-related mechanisms, can force exhausted cells into an irreversible exhaustion-specific and developmentally fixed chromatin state, which cannot be remodeled by anti-PD-1 treatment.

Further studies also provide information on the dynamic regulation of methylation patterns during virus-induced T cell differentiation (64, 65). For instance, in naïve CD8^+^ T cells, genes associated with effector functions are marked with H3K4me3 indicating that effector function related genes are repressed in naïve CD8^+^ T cells. Upon activation, the same genes acquire chromatin marks that are related to an active transcription (64, 65).

In addition to the regulation of chromatin marks of promoters or enhancers, which are characterized by H3K4me1 and H3K27ac, are also differentially remodeled during T cell activation and differentiation (59, 66). In studies by Kakaradov et al., around 50,000 enhancers were identified and about 50% of them were found to be shared between all stages of T cell activation and differentiation, whereas the other half of enhancers was either gained or lost depending on the state of the cells suggesting that there is a dynamic regulation of enhancers similar to the other epigenetic modifications of T cells during differentiation (66). According to single-cell sequencing studies, in which individual CD8^+^ T cells were analyzed during an acute LCMV infection in mice, the differentiation of terminal effector cells was initiated by an early burst of transcriptional activity followed by a refinement of epigenetic silencing of transcripts related to memory lymphocytes through H3K27me3 and Ezh2, which is the catalytic subunit of polycomb repressive complex 2 (PRC2) (67).

## The Effects of Tumor Microenvironment on T Cell Metabolism and Epigenetics

Chronic infections and cancer share common properties in terms of CD8^+^ T cell functions. In both cases, antigen specific CD8^+^ T cells progress into the so-called exhausted state due to continuous antigen exposure resulting in increased expression of exhaustion markers. Therefore, research on chronic infection models in mice as well as patient samples contributed to the development of anti-cancer therapies, which target T cell exhaustion such as anti-PD-1 or adoptive T cell therapies (68). Although the reprogramming of cellular metabolism and changes of the epigenetic landscape of CD8^+^ T cells have been intensively studied during chronic infections, these mechanisms are still poorly understood in tumor infiltrating lymphocytes (TILs). Although overlapping features of exhausted CD8^+^ T cells can be observed both in chronic infection and cancer models, the specific metabolic environment in tumors represents an additional, unique factor shaping T cell activation and differentiation via the specific supply provision of metabolites and various secreted signaling molecules (Figure 2B).

The metabolism of tumor cells is altered compared to normal cells, which metabolize glucose through OXPHOS. Instead, highly proliferating tumor cells use glycolysis pathway to metabolize glucose in order to supply the anabolic products needed for rapid cell growth and division. This metabolic alteration of tumor cells was characterized by Otto Warburg almost a century ago and is now considered as a hallmark of cancer (69). However, this phenomenon is only a portion of the unique tumor cell metabolism. In addition to glucose metabolism, lipid, amino acid, and adenosine metabolism are also altered in tumor cells to meet their high-energy demands (70). For instance, HIF1α in tumor cells upregulates the expression of CD73, which is located on the surface of many tumor cells and which is responsible for the conversion of adenosine monophosphate to adenosine resulting in increased adenosine concentrations in TME (71). Similarly, HIF1α also regulates genes critical for the lipid metabolism such as COX2 whose overexpression is associated with poor prognosis in several solid tumor cancers (72). Glutamine metabolism is also altered in tumor cells, that are known as glutamine traps since they have higher levels of glutamine uptake (70).

TILs are mostly non-functional or exhausted due to the highly immunosuppressive TME. The depletion of glucose in TME by tumor cells represents one “exhausting” mechanism and results in a decrease of aerobic glycolysis in TILs and decreased phosphoenolpyruvic acid (PEP) production that is a crucial metabolite participating in TCR-dependent activation of calcium pathways like SOCE and NFAT signaling in T cells (73, 74), which is critical for proper anti-tumor functions (73). Additionally, due to high lactate production of tumor cells, the acidity of TME increases, resulting in the inhibition of key T cell responses such as proliferation after activation and effector cytokine production by CD8^+^ T cells (70, 75). Similarly, due to the hypoxic environment of the tumors, HIF1α upregulates the expression of PD-1-ligand leading to inhibition of CD8^+^ T-cell mediated cytotoxicity (76).

The epigenetic landscapes of tumor infiltrating CD8^+^ T cells are not well understood. In a recent study, Philipp et al. defined the chromatin dynamics of tumor-specific dysfunctional cells over the course of tumorigenesis (77). They observed that naïve tumor-specific T cells that encounter their antigen firstly acquire a plastic, dysfunctional chromatin state that can be remodeled. Later, the same cells differentiate into a fixed dysfunctional chromatin state, which cannot be remodeled or rescued anymore during the progression of large established tumors. In addition, human dysfunctional tumor specific T cells with high PD-1 expression share many core elements with these mouse models (77). Interestingly, tumor-specific memory T cells also differentiate into the same fixed dysfunctional chromatin state suggesting that regardless of the initial chromatin states of the cells, continuous antigen exposure in tumors can overwrite this fixed dysfunctional chromatin state (77).

## Conclusion

Since the manipulation of metabolic pathways *in vivo* is very challenging, so far mostly *in vitro* systems served in this field, to provide mechanistic information to reveal the regulation of T cell metabolism in a controlled environment. However, the field is still in need of experimental models that are able to better provide the physiological context of changing T cell environments such as nutrient availability, interaction between different cell types and cytokine milieu to fully investigate the role of metabolism during T cell activation and differentiation.

The link between epigenetic changes and cellular metabolism has been intensively studied in cancer cells. However, the role of metabolism on T cell function and differentiation has only recently been characterized despite growing knowledge about the connection of T cell epigenetics during differentiation and function by using genome-wide mapping of accessible chromatin sites. However, it still remains elusive how metabolites regulate the epigenome of T cells in a gene-specific manner.

Although this interplay between tumor cells and the epigenetic regulation of TILs remains elusive, a better understanding of the epigenetic regulation of exhaustion and the metabolic fitness of TILs might hold potential to improve current cancer therapies such as checkpoint blockade and adoptive T cell therapies. The relationship between the unique metabolism in TME and how it affects the epigenome of TILs might help to find ways to rescue their exhausted phenotype via epigenome-targeting pharmacological drugs to boost immune responses against tumor cells.

## Author Contributions

CY designed, wrote, and revised the manuscript. BS revised the manuscript. CW and RG designed and revised the manuscript.

### Conflict of Interest

The authors declare that the research was conducted in the absence of any commercial or financial relationships that could be construed as a potential conflict of interest. The reviewer WE and handling editor declared their shared affiliation at the time of review.

## References

[1] Best JA Blair DA Knell J Yang E Mayya V Doedens A. Transcriptional insights into the CD8+ T cell response to infection and memory T cell formation. Nat Immunol. (2013) 14:404–12. 10.1038/ni.2536 23396170 PMC3689652

[2] Kannan A Huang W Huang F August A. Signal transduction via the T cell antigen receptor in naïve and effector/memory T cells. Int J Biochem Cell Biol. (2012) 44:2129–34. 10.1016/j.biocel.2012.08.023 22981631 PMC3532926

[3] Lu C Thompson CB. Metabolic regulation of epigenetics. Cell Metab. (2012) 16:9–17. 10.1016/j.cmet.2012.06.001 22768835 PMC3392647

[4] Sadakierska-Chudy A Filip M A comprehensive view of the epigenetic landscape. Part II: histone post-translational modification, nucleosome level, and chromatin regulation by ncRNAs. Neurotox Res. (2014) 27:172–97. 10.1007/s12640-014-9508-6 PMC4300421 25516120

[5] Burgess RJ Zhang Z. Histone chaperones in nucleosome assembly and human disease. Nat Struct Mol Biol. (2013) 20:14–22. 10.1038/nsmb.2461 23288364 PMC4004355

[6] Zhang T Cooper S Brockdorff N. The interplay of histone modifications – writers that read. EMBO Rep. (2015) 16:1467–81. 10.15252/embr.201540945 26474904 PMC4641500

[7] Henikoff S Smith MM. Histone variants and epigenetics. Cold Spring Harb Perspect Biol. (2015) 7:a019364. 10.1101/cshperspect.a019364 25561719 PMC4292162

[8] Gregory PD Wagner K Hörz W. Histone acetylation and chromatin remodeling. Exp Cell Res. (2001) 265:195–202. 10.1006/excr.2001.5187 11302684

[9] Eberharter A. Histone acetylation: a switch between repressive and permissive chromatin: second in review series on chromatin dynamics. EMBO Rep. (2002) 3:224–9. 10.1093/embo-reports/kvf053 11882541 PMC1084017

[10] Roth SY Denu JM Allis CD. Histone acetyltransferases. Ann Rev Biochem. (2001) 70:81–120. 10.1146/annurev.biochem.70.1.81 11395403

[11] Marmorstein R Zhou MM. Writers and readers of histone acetylation: structure, mechanism, and inhibition. Cold Spring Harb Perspect Biol. (2014) 6:a018762. 10.1101/cshperspect.a018762 24984779 PMC4067988

[12] Pietrocola F Galluzzi L Bravo-San P José M Madeo F Kroemer G. Acetyl coenzyme A: a central metabolite and second messenger. Cell Metab. (2015) 21:805–21. 10.1016/j.cmet.2015.05.014 26039447

[13] Seto E Yoshida M. Erasers of histone acetylation: the histone deacetylase enzymes. Cold Spring Harb Perspect Biol. (2014) 6:a018713. 10.1101/cshperspect.a018713 24691964 PMC3970420

[14] Suraweera A O'Byrne KJ Richard DJ. Combination therapy with histone deacetylase inhibitors (HDACi) for the treatment of cancer: achieving the full therapeutic potential of HDACi. Front Oncol. (2018) 8:92. 10.3389/fonc.2018.00092 29651407 PMC5884928

[15] Akimova T Beier UH Liu Y Wang L Hancock WW. Histone/protein deacetylases and T-cell immune responses. Blood. (2012) 119:2443–51. 10.1182/blood-2011-10-292003 22246031 PMC3311271

[16] Roy S Packman K Jeffrey R Tenniswood M Histone deacetylase inhibitors differentially stabilize acetylated p53 and induce cell cycle arrest or apoptosis in prostate cancer cells. Cell Death Differ. (2005) 12:482–91. 10.1038/sj.cdd.4401581 15746940

[17] de Zoeten EF Wang L Butler K Beier UH Akimova T Sai H. Histone deacetylase 6 and heat shock protein 90 control the functions of Foxp3+ T-regulatory cells. Mol Cell Biol. (2011) 31:2066–78. 10.1128/MCB.05155-11 21444725 PMC3133361

[18] de Zoeten EF Wang L Sai H Dillmann WH Hancock WW. Inhibition of HDAC9 increases T regulatory cell function and prevents colitis in mice. Gastroenterology. (2010) 138:583–94. 10.1053/j.gastro.2009.10.037 19879272 PMC3369426

[19] Luu M Weigand K Wedi F Breidenbend C Leister H Pautz S. Regulation of the effector function of CD8+ T cells by gut microbiota-derived metabolite butyrate. Sci Rep. (2018) 8:14430. 10.1038/s41598-018-32860-x 30258117 PMC6158259

[20] Kasler HG Lim HW Mottet D Collins AM Lee IS Verdin E. Nuclear export of histone deacetylase 7 during thymic selection is required for immune self-tolerance. EMBO J. (2012) 31:4453–65. 10.1038/emboj.2012.295 23103766 PMC3512390

[21] Kasler HG Verdin E. Histone deacetylase 7 functions as a key regulator of genes involved in both positive and negative selection of thymocytes. Mol Cell Biol. (2007) 27:5184–200. 10.1128/MCB.02091-06 17470548 PMC1951960

[22] Kasler HG Young BD Mottet D Lim HW Collins AM Olson EN. Histone deacetylase 7 regulates cell survival and TCR signaling in CD4/CD8 double-positive thymocytes. J Immunol. (2011) 186:4782–93. 10.4049/jimmunol.1001179 21398603

[23] Navarro MN Goebel J Feijoo-Carnero C Morrice N Cantrell DA. Phosphoproteomic analysis reveals an intrinsic pathway for the regulation of histone deacetylase 7 that controls the function of cytotoxic T lymphocytes. Nat Immunol. (2011) 12:352–61. 10.1038/ni.2008 21399638 PMC3110993

[24] Xiao H Jiao J Wang L O'Brien S Newick K Wang L-CS. HDAC5 controls the functions of Foxp3+T-regulatory and CD8+T cells. Int J Cancer. (2016) 138:2477–86. 10.1002/ijc.29979 26704363 PMC5484398

[25] Tsuji G Okiyama N Villarroel VA Katz SI. Histone deacetylase 6 inhibition impairs effector CD8 T-cell functions during skin inflammation. J Allergy Clin Immunol. (2015) 135:1228–39. 10.1016/j.jaci.2014.10.002 25458911 PMC4426217

[26] Laino AS Betts BC Veerapathran A Dolgalev I Sarnaik A Quayle SN. HDAC6 selective inhibition of melanoma patient T-cells augments anti-tumor characteristics. J ImmunoTher Cancer. (2019) 7:33. 10.1186/s40425-019-0517-0 30728070 PMC6366050

[27] Núñez-Andrade N Iborra S Trullo A Moreno-Gonzalo O Calvo E Catalán E. HDAC6 regulates the dynamics of lytic granules in cytotoxic T lymphocytes. J Cell Sci. (2016) 129:1305–11. 10.1242/jcs.180885 26869226 PMC5023047

[28] Stengel KR Zhao Y Klus NJ Kaiser JF Gordy LE Joyce S. Histone deacetylase 3 is required for efficient T cell development. Mol Cell Biol. (2015) 35:3854–65. 10.1128/MCB.00706-15 26324326 PMC4609739

[29] Dovey OM Foster CT Conte N Edwards SA Edwards JM Singh R. Histone deacetylase 1 and 2 are essential for normal T-cell development and genomic stability in mice. Blood. (2013) 121:1335–44. 10.1182/blood-2012-07-441949 23287868 PMC3836254

[30] Heideman MR Wilting RH Yanover E Velds A de Jong J Kerkhoven RM. Dosage-dependent tumor suppression by histone deacetylases 1 and 2 through regulation of c-Myc collaborating genes and p53 function. Blood. (2013) 121:2038–50. 10.1182/blood-2012-08-450916 23327920 PMC3596963

[31] Tschismarov R Firner S Gil-Cruz C Göschl L Boucheron N Steiner G. HDAC1 controls CD8+ T cell homeostasis and antiviral response. PLoS ONE. (2014) 9:e110576. 10.1371/journal.pone.0110576 25333902 PMC4204873

[32] Kelly G. A review of the sirtuin system, its clinical implications, and the potential role of dietary activators like resveratrol: part 1. Altern Med Rev. (2010) 15:245–63. 21155626

[33] Craveiro M Cretenet G Mongellaz C Matias MI Caron O de Lima MCP. Resveratrol stimulates the metabolic reprogramming of human CD4+T cells to enhance effector function. Sci Signal. (2017) 10:eaal3024. 10.1126/scisignal.aal3024 29042482

[34] Tran TQ Lowman XH Kong M. Molecular pathways: metabolic control of histone methylation and gene expression in cancer. Clin Cancer Res. (2017) 23:4004–9. 10.1158/1078-0432.CCR-16-2506 28404599 PMC5553983

[35] Jeng MY Hull PA Fei M Kwon H-S Tsou C-L Kasler H. Metabolic reprogramming of human CD8+ memory T cells through loss of SIRT1. J Exp Med. (2017) 215:51–62. 10.1084/jem.20161066 29191913 PMC5748845

[36] Chisolm DA Weinmann AS. Connections between metabolism and epigenetics in programming cellular differentiation. Ann Rev Immunol. (2018) 36:221–46. 10.1146/annurev-immunol-042617-053127 29328786

[37] Ma EH Bantug G Griss T Condotta S Johnson RM Samborska B Serine is an essential metabolite for effector T cell expansion. Cell Metab. (2017) 25:482 10.1016/j.cmet.2016.12.011 28178570

[38] Narlikar GJ Sundaramoorthy R Owen-Hughes T. Mechanisms and functions of ATP-dependent chromatin-remodeling enzymes. Cell. (2013) 154:490–503. 10.1016/j.cell.2013.07.011 23911317 PMC3781322

[39] Phan AT Goldrath AW Glass CK. Metabolic and epigenetic coordination of T cell and macrophage immunity. Immunity. (2017) 46:714–29. 10.1016/j.immuni.2017.04.016 28514673 PMC5505665

[40] Blagih J Coulombe F Vincent EE Dupuy F Galicia-Vázquez G Yurchenko E. The energy sensor AMPK regulates T cell metabolic adaptation and effector responses *in vivo*. Immunity. (2015) 42:41–54. 10.1016/j.immuni.2014.12.030 25607458

[41] Rao E Zhang Y Zhu G Hao J Persson X-MT Egilmez NK. Deficiency of AMPK in CD8^+^ T cells suppresses their anti-tumor function by inducing protein phosphatase-mediated cell death. Oncotarget. (2015) 6:7944–58. 10.18632/oncotarget.3501 25760243 PMC4480727

[42] Metallo CM Vander H Matthew G. Understanding metabolic regulation and its influence on cell physiology. Mol Cell. (2013) 49:388–98. 10.1016/j.molcel.2013.01.018 23395269 PMC3569837

[43] Kaech SM Wherry EJ. Heterogeneity and cell-fate decisions in effector and memory CD8+ T cell differentiation during viral infection. Immunity. (2007) 27:393–405. 10.1016/j.immuni.2007.08.007 17892848 PMC3431921

[44] Vaeth M Maus M Klein-Hessling S Freinkman E Yang J Eckstein M. Store-operated Ca2+ entry controls clonal expansion of T cells through metabolic reprogramming. Immunity. (2017) 47:664–679.e666. 10.1016/j.immuni.2017.09.003 29030115 PMC5683398

[45] Buck MD O'Sullivan D Klein G Ramon I Curtis JD Chang C-H. Mitochondrial dynamics controls T cell fate through metabolic programming. Cell. (2016) 166:63–76. 10.1016/j.cell.2016.05.035 27293185 PMC4974356

[46] van der Windt GJW Everts B Chang C-H Curtis JD Freitas TC Amiel E. Mitochondrial respiratory capacity is a critical regulator of CD8+ T cell memory development. Immunity. (2012) 36:68–78. 10.1016/j.immuni.2011.12.007 22206904 PMC3269311

[47] Chang C-H Curtis JD Maggi LB Faubert B Villarino AV O'Sullivan D. Posttranscriptional control of T cell effector function by aerobic glycolysis. Cell. (2013) 153:1239–51. 10.1016/j.cell.2013.05.016 23746840 PMC3804311

[48] Buck MD O'Sullivan D Pearce EL. T cell metabolism drives immunity. J Exp Med. (2015) 212:1345–60. 10.1084/jem.20151159 26261266 PMC4548052

[49] Raud B Roy DG Divakaruni AS Tarasenko TN Franke R Ma EH. Etomoxir actions on regulatory and memory T cells are independent of Cpt1a-mediated fatty acid oxidation. Cell Metab. (2018) 28:504–15.e507. 10.1016/j.cmet.2018.06.002 30043753 PMC6747686

[50] Divakaruni AS Hsieh WY Minarrieta L Duong TN Kim KKO Desousa BR. Etomoxir inhibits macrophage polarization by disrupting CoA homeostasis. Cell Metab. (2018) 28:490–503.e497. 10.1016/j.cmet.2018.06.001 30043752 PMC6125190

[51] Delgoffe GM Powell JD. Feeding an army: the metabolism of T cells in activation, anergy, and exhaustion. Mol Immunol. (2015) 68:492–6. 10.1016/j.molimm.2015.07.026 26256793 PMC4837657

[52] Staron MM Gray SM Marshall HD Parish IA Chen JH Perry CJ. The transcription factor FoxO1 sustains expression of the inhibitory receptor PD-1 and survival of antiviral CD8+ T cells during chronic infection. Immunity. (2014) 41:802–14. 10.1016/j.immuni.2014.10.013 25464856 PMC4270830

[53] Bengsch B Johnson AL Kurachi M Odorizzi PM Pauken KE Attanasio J. Bioenergetic insufficiencies due to metabolic alterations regulated by the inhibitory receptor PD-1 are an early driver of CD8 + T cell exhaustion. Immunity. (2016) 45:358–73. 10.1016/j.immuni.2016.07.008 27496729 PMC4988919

[54] Savan R. Post-transcriptional regulation of interferons and their signaling pathways. J Interferon Cytokine Res. (2014) 34:318–29. 10.1089/jir.2013.0117 24702117 PMC4015472

[55] Peng M Yin N Chhangawala S Xu K Leslie CS Li MO. Aerobic glycolysis promotes T helper 1 cell differentiation through an epigenetic mechanism. Science. (2016) 354:481–4. 10.1126/science.aaf6284 27708054 PMC5539971

[56] Tyrakis PA Palazon A Macias D Lee KL Phan AT Veliça P. S-2-hydroxyglutarate regulates CD8+ T-lymphocyte fate. Nature. (2016) 540:236–41. 10.1038/nature20165 27798602 PMC5149074

[57] Scharer CD Bally APR Gandham B Boss JM. Cutting edge: chromatin accessibility programs CD8 T cell memory. J Immunol. (2017) 198:2238–43. 10.4049/jimmunol.1602086 28179496 PMC5341694

[58] Scott-Browne JP López-Moyado IF Trifari S Wong V Chavez L Rao A. Dynamic changes in chromatin accessibility occur in CD8 + T cells responding to viral infection. Immunity. (2016) 45:1327–40. 10.1016/j.immuni.2016.10.028 27939672 PMC5214519

[59] Yu B Zhang K Milner JJ Toma C Chen R Scott-Browne JP Epigenetic landscapes reveal transcription factors that regulate CD8+ T cell differentiation. Nat Immunol. (2017) 18:573–82. 10.1038/ni.3706 28288100 PMC5395420

[60] Sen DR Kaminski J Barnitz RA Kurachi M Gerdemann U Yates KB. The epigenetic landscape of T cell exhaustion. Science. (2016) 354:1165–9. 10.1126/science.aae0491 27789799 PMC5497589

[61] Alfei F Kanev K Hofmann M Wu M Ghoneim HE Roelli P. TOX reinforces the phenotype and longevity of exhausted T cells in chronic viral infection. Nature. (2019) 571:265–9. 10.1038/s41586-019-1326-9 31207605

[62] Khan O Giles JR McDonald S Manne S Ngiow SF Patel KP. TOX transcriptionally and epigenetically programs CD8+ T cell exhaustion. Nature. (2019) 571:211–8. 10.1038/s41586-019-1325-x 31207603 PMC6713202

[63] Seo H Chen J González-Avalos E Samaniego-Castruita D Das A Wang YH. TOX and TOX2 transcription factors cooperate with NR4A transcription factors to impose CD8+ T cell exhaustion. Proc Natl Acad Sci USA. (2019) 116:12410–5. 10.1073/pnas.1905675116 31152140 PMC6589758

[64] Araki Y Wang Z Zang C Wood WH Schones D Cui K. Genome-wide analysis of histone methylation reveals chromatin state-based regulation of gene transcription and function of memory CD8+ T cells. Immunity. (2009) 30:912–25. 10.1016/j.immuni.2009.05.006 19523850 PMC2709841

[65] Russ BE Olshanksy M Smallwood HS Li J Denton AE Prier JE. Distinct epigenetic signatures delineate transcriptional programs during virus-specific CD8+ T cell differentiation. Immunity. (2014) 41:853–65. 10.1016/j.immuni.2014.11.001 25517617 PMC4479393

[66] Kakaradov B Arsenio J Widjaja CE He Z Aigner S Metz PJ. Early transcriptional and epigenetic regulation of CD8+ T cell differentiation revealed by single-cell RNA sequencing. Nat Immunol. (2017) 18:422–32. 10.1038/ni.3688 28218746 PMC5360497

[67] Gray SM Amezquita RA Guan T Kleinstein SH Kaech SM. Polycomb repressive complex 2-mediated chromatin repression guides effector CD8 + T cell terminal differentiation and loss of multipotency. Immunity. (2017) 46:596–608. 10.1016/j.immuni.2017.03.012 28410989 PMC5457165

[68] Vance RE Eichberg MJ Portnoy DA Raulet DH. Listening to each other: infectious disease and cancer immunology. Sci Immunol. (2017) 2:eaai9339. 10.1126/sciimmunol.aai9339 28783669 PMC5927821

[69] Warburg O. The metabolism of tumors in the body. J Gen Physiol. (1927) 8:519–30. 10.1085/jgp.8.6.519 19872213 PMC2140820

[70] Renner K Singer K Koehl GE Geissler EK Peter K Siska PJ. Metabolic hallmarks of tumor and immune cells in the tumor microenvironment. Front Immunol. (2017) 8:248. 10.3389/fimmu.2017.00248 28337200 PMC5340776

[71] Kaidi A Qualtrough D Williams AC Paraskeva C. Direct transcriptional up-regulation of cyclooxygenase-2 by hypoxia-inducible factor (HIF)-1 promotes colorectal tumor cell survival and enhances HIF-1 transcriptional activity during hypoxia. Cancer Res. (2006) 66:6683–91. 10.1158/0008-5472.CAN-06-0425 16818642

[72] Baba Y Nosho K Shima K Irahara N Chan AT Meyerhardt JA. HIF1A overexpression is associated with poor prognosis in a cohort of 731 colorectal cancers. Am J Pathol. (2010) 176:2292–301. 10.2353/ajpath.2010.090972 20363910 PMC2861094

[73] Ho P-C Bihuniak JD Macintyre AN Staron M Liu X Amezquita R. Phosphoenolpyruvate is a metabolic checkpoint of anti-tumor T cell responses. Cell. (2015) 162:1217–28. 10.1016/j.cell.2015.08.012 26321681 PMC4567953

[74] Weidinger C Shaw PJ Feske S STIM 1 and STIM 2-mediated Ca2+ influx regulates antitumour immunity by CD8+ T cells. EMBO Mol Med. (2013) 5:1311–21. 10.1002/emmm.201302989 23922331 PMC3799488

[75] Fischer K Hoffmann P Voelkl S Meidenbauer N Ammer J Edinger M. Inhibitory effect of tumor cell–derived lactic acid on human T cells. Blood. (2007) 109:3812–9. 10.1182/blood-2006-07-035972 17255361

[76] Noman MZ Desantis G Janji B Hasmim M Karray S Dessen P. PD-L1 is a novel direct target of HIF-1α, and its blockade under hypoxia enhanced MDSC-mediated T cell activation. J Exp Med. (2014) 211:781–90. 10.1084/jem.20131916 24778419 PMC4010891

[77] Philip M Fairchild L Sun L Horste EL Camara S Shakiba M. Chromatin states define tumour-specific T cell dysfunction and reprogramming. Nature. (2017) 545:452–6. 10.1038/nature22367 28514453 PMC5693219

